# Therapeutic potential of mature adipocyte-derived dedifferentiated fat cells for inflammatory bowel disease

**DOI:** 10.1007/s00383-020-04681-5

**Published:** 2020-05-24

**Authors:** Shigeki Ishioka, Takashi Hosokawa, Taro Ikeda, Noriyoshi Konuma, Hide Kaneda, Kensuke Ohashi, Takeshi Furuya, Takayuki Masuko, Hiroaki Taniguchi, Koichiro Kano, Tsugumichi Koshinaga, Taro Matsumoto

**Affiliations:** 1 Department of Pediatric Surgery, Nihon University School of Medicine, Tokyo, 173-8610 Japan; 2 Department of Surgery, Saitama Medical Center, Jichi Medical University, Saitama, 330-8503 Japan; 3 Pediatric Surgery and Urology, Ibaraki Children’s Hospital, Mito, 311-4145 Japan; 4 Institute of Genetics and Animal Breeding of the Polish Academy of Sciences, 05-552, Magdalenka, Poland; 5 Laboratory of Cell and Tissue Biology, College of Bioresource Science, Nihon University, Fujisawa, 252-0880 Japan; 6 Division of Cell Regeneration and Transplantation, Department of Functional Morphology, Nihon University School of Medicine, 30-1 Ohyaguchikami-cho, Itabashi-ku, Tokyo, 173-8610 Japan

**Keywords:** Adipocytes, Inflammatory bowel diseases, Mesenchymal stromal cells, Regenerative medicine

## Abstract

**Purpose:**

Our previous studies demonstrated that mature adipocyte-derived dedifferentiated fat (DFAT) cells possess similar multipotency as mesenchymal stem cells. Here, we examined the immunoregulatory potential of DFAT cells in vitro and the therapeutic effect of DFAT cell transplantation in a mouse inflammatory bowel disease (IBD) model.

**Methods:**

The effect of DFAT cell co-culture on T cell proliferation and expression of immunosuppression-related genes in DFAT cells were evaluated. To create IBD, CD4^+^CD45RB^high^ T cells were intraperitoneally injected into SCID mice. One week later, DFAT cells (1 × 10^5^, DFAT group) or saline (Control group) were intraperitoneally injected. Subsequently bodyweight was measured every week and IBD clinical and histological scores were evaluated at 5 weeks after T cell administration.

**Results:**

The T cell proliferation was inhibited by co-cultured DFAT cells in a cell density-dependent manner. Gene expression of *TRAIL*, *IDO1*, and *NOS2* in DFAT cells was upregulated by TNFα stimulation. DFAT group improved IBD-associated weight loss, IBD clinical and histological scores compared to Control group.

**Conclusion:**

DFAT cells possess immunoregulatory potential and the cell transplantation promoted recovery from colon damage and improved clinical symptoms in the IBD model. DFAT cells could play an important role in the treatment of IBD.

## Background

Inflammatory bowel disease (IBD), including Crohn’s disease and ulcerative colitis, is a family of chronic, idiopathic, and relapsing diseases characterized by dysfunction of mucosal T cells and altered cellular inflammation that ultimately lead to damage of the distal small intestine and colonic mucosa [1]. Although the precise mechanisms that trigger IBD remain unknown, research points to the involvement of environmental factors, genetics, microbial factors, and mucosal immune defects [2]. Currently, the widely accepted hypothesis for the etiology of IBD is that of a disturbed interaction between the host immune system and the commensal microflora and other luminal antigens. This interaction, in turn, leads to ongoing mucosal inflammation [3]. In the host immune system, a loss of balance between proinflammatory T helper cells such as Th1 and Th17 cells and regulatory T cells play a central role in the pathogenesis of IBD [4].

Interestingly, mesenchymal stem cells (MSCs) have gained attention as an effective therapeutic agent for tissue repair [5, 6]. MSCs were shown to modulate both the innate and adaptive immune systems [7–10]. These cells may also inhibit the function of various immune cells, including dendritic cells, T cells, B cells, and natural killer cells. The immunomodulatory and anti-inflammatory properties of MSCs have been elucidated in IBD models [11–16]. Furthermore, MSCs have recently shown great promise in treating IBD in clinical studies [17]. The mechanisms that drive the immunomodulatory effects of MSCs include direct cellular contact and the secretion of a wide spectrum of soluble factors, such as transforming growth factor-β (TGF-β), prostaglandin E_2_ (PGE_2_), indoleamine-2,3-dioxygenase (IDO), nitric oxide (NO), and hepatocyte growth factor (HGF) [18]. Nonetheless, the quantity of MSCs obtained from donors is often much less than the amount required for tissue regeneration. Moreover, the technique for isolating MSCs is relatively invasive.

More specifically, lipid-filled adipocytes isolated from adipose tissue can be dedifferentiated into fibroblast-like cells using ceiling culture method [19]. These adipocyte-derived fibroblast-like cells can proliferate rapidly and be redifferentiated into mature adipocytes both in vitro and in vivo. Accordingly, these cells possess characteristics similar to adipogenic progenitor cells. Our group has, therefore, named these cells dedifferentiated fat (DFAT) cells [20]. DFAT cells can be differentiated into adipogenic, osteogenic, chondrogenic, and myogenic lineages under certain cell culture conditions. Additionally, analysis with flow cytometry demonstrates that DFAT cells are relatively homogeneous when compared to MSCs [21]. Since fat tissue is usually abundant and easily accessible at nearly every age, DFAT cells are an attractive resource for cell-based therapies targeting disease including IBD. To date, little is known about the effect of DFAT cells on T cell function and T cell-related diseases including IBD. The aim of this study is to elucidate whether DFAT cells can modulate T cell proliferation in vitro and subsequently improve the intestinal inflammation found in a mouse T cell-transfer model of IBD.

## Materials and methods

### Animals

The 9- to 11-week-old female BALB/c mice and 8- to 10-week-old female severe combined immunodeficiency (SCID) mice were purchased from Oriental Yeast Co., Ltd. (Tokyo, Japan). All animal experiments were performed after receiving approval from the Animal Research and Care Committee of the Nihon University School of Medicine.

### Cell isolation and culture

Samples of human subcutaneous adipose tissue were obtained from patients undergoing surgery in the Departments of Pediatric Surgery of Nihon University Itabashi Hospital (Tokyo, Japan). The patients gave written informed consent, and the Ethics Committee of Nihon University School of Medicine approved the study. Preparation of DFAT cells using the ceiling culture was described previously [21]. Briefly, fat tissue (approximately 1 g) was cut into small pieces and digested with 0.1% type I collagenase solution (Koken Co., Ltd., Tokyo, Japan) at 37 °C for 1 h. Neutralized cells were filtered and centrifuged at 135 g for 3 min, followed by collecting the floating cell layer containing adipocytes. The isolated adipocytes were washed with phosphate-buffered saline (PBS), plated in 25-cm^2^ culture flasks (NUNC, Roskilde, Denmark) filled completely with Dulbecco’s modified Eagle’s medium (DMEM; Invitrogen, Carlsbad, CA, USA) containing 20% fetal bovine serum (FBS; JRH Bioscience, Lenexa, KS, USA) with 5 × 10^4^ cells per flask. The cells were incubated at 37 °C with 5% CO_2_ for 7 days. The adipocytes immediately floated up and subsequently adhered to the top ceiling surface of the flask within 2–3 days of the culture. On day 7, the flasks were inverted after remove media and adhered cells were cultured in 5 ml DMEM containing 20% FBS. The medium was changed every 4 days until the cells reached confluence. The cells were passaged by standard methods of trypsinization and were used for experiments at passage 1. The preparation of BALB/c mouse DFAT cells from adipose tissue was performed according to the preparation of human DFAT cells.

### T cell proliferation assay

Mouse T cell proliferation assay was performed as described by Kruisbeek et al. [22]. Briefly, CD3^+^ cells were isolated from the spleen of a BALB/c mouse using Dynabeads Untouched Mouse T Cells Kit (Invitrogen). Mouse DFAT cells were cultured overnight in a flat-bottom 96-well plate (BD Falcon, Franklin Lakes, NJ, USA) at the concentrations of 1 × 10^5^, 2.5 × 10^5^, and 5 × 10^5^. The mouse CD3^+^ cells (5 × 10^4^/well) were then added and cultured for 24 h in RPMI 1640 medium (Invitrogen) supplemented with 10% FBS without (unstimulated) or with 5 μl/well anti-CD3/28 antibody beads (Invitrogen) and 20 ng/ml mouse interleukin-2 (IL-2, Cell Signaling Technologies, Danvers, MA, USA). Cell proliferation was evaluated for 24 h as bromodeoxyuridine (BrdU) incorporation using Cell Proliferation ELISA Biotrak Version 2 ELISA (GE Healthcare, Buckinghamshire, UK) according to the manufacturer’s instructions.

### Real-time reverse transcription polymerase chain reaction (RT-PCR)

Mouse DFAT cells (1 × 10^5^/well) were stimulated or not for 48 h with mouse 750 IU/ml of interferon (IFN)β (Abcam, Cambridge, UK), 30 IU/ml of mouse IFNγ (BD Biosciences, San Jose, CA), or 10 ng/ml of mouse tumor necrosis factor (TNF)α (Cedarlane, Burlington, Canada). Total RNA (1 μg) was isolated from cell pellets using an RNeasy Mini Kit (Qiagen, Hilden, Germany). First-strand cDNA synthesis was performed using a High-Capacity cDNA Reverse Transcription Kit (Applied Biosystems, Foster City, CA, USA). mRNA of the genes of interest was quantitated by real-time RT-PCR (7300 Real-Time PCR System, Applied Biosystems) using SYBR Green Master Mix (Applied Biosystems). Primers sequences were as follows: *TNF related apoptosis-inducing ligand* (*TRAIL*): forward, 5′-ATGGCCTGGCTGTAGAAACCT-3′ and reverse, 5′-GAAACACCGAAAGTGTCTGTGG-3′; *nitric oxide synthase 2* (*NOS2*): forward, 5′-GCCACCAACAATGGCAACA-3′ and reverse, 5′-CGTACCGGATGAGCTGTGAATT-3′; *prostaglandin-endoperoxide synthase 2* (*PTGS2*): forward, 5′-AGGCAAAGCTGAAGGCAGAGA-3′ and reverse, 5′-AAAAACCGAAGAGCTCGGAGG-3′; *hepatocyte growth factor* (*HGF*): forward, 5′-GCTCCAGCTTCCAAATTGCA-3′ and reverse, 5′-CAGAAGTTTGGTCCCCCACAT-3′; *indoleamine 2,3-dioxygenase 1* (*IDO1*): forward, 5′-GACTTTGTGGACCCAGACACGT-3′ and reverse, 5′-ACCCCCTCATACAGCAGACCTT-3′; and *glyceraldehyde-3-phosphate dehydrogenase* (*GAPDH*): forward, 5′-GCAAAGTGGAGATTGTTGCCAT-3′ and reverse, 5′-CCTTGACTGTGCCGTTGAATTT-3′. Differences in gene expression were assessed by the comparative Ct method with *GAPDH* as the endogenous control.

### Effect of DFAT cell transplantation in a mouse model of IBD

Induction of colitis by adoptive transfer of CD4^+^CD45RB^high^ T cells into SCID mice was performed essentially as described previously [23]. CD4^+^CD45RB^high^ T cells (3 × 10^5^ cells in 200 μl PBS) isolated from a BALB/c mouse spleen were intraperitoneally injected into SCID mice (*n* = 12). One week later, human DFAT cells (1 × 10^5^/200 μl PBS, DFAT group, *n* = 6) or 200 μl PBS (Control group, *n* = 6) were intraperitoneally injected into the IBD model mice. Body weights of each group were measured every week to evaluate induced colitis. The rate of body weight loss was calculated based on the body weights before T cell administration. The mice were sacrificed at 5 weeks after T cell administration, and after immobilization with 4% paraformaldehyde, the colon was taken and the IBD clinical score [24] was evaluated in each group. The colon was then embedded in paraffin, and samples were sectioned at 4 μm. Samples were then deparaffinized and stained with hematoxylin and eosin. The samples were examined under a BX51 microscope (Olympus, Tokyo, Japan) and photographed using a DP20-5 microscope digital camera (Olympus). Intestinal inflammation was evaluated using the IBD histological score [24] in each group.

### Statistical analysis

All results are expressed as mean ± standard deviation (SD) or mean ± standard error (SE). One-way analysis of variance (ANOVA) in combination with Tukey’s post hoc test were used for comparison of BrdU incorporation between the groups. Wilcoxon test was used for comparison of % of body weight loss between the groups. Mann–Whitney *U* test was used for comparison of clinical and histological scores between the groups. GraphPad Prism (ver 5.0, GraphPad Software, La Jolla, CA, USA) was used for the statistical analysis. Statistical significance was defined as *P* < 0.05.

## Results

### Impact of DFAT cells on CD3^+^ T cell proliferation in vitro

**Fig. 1:**
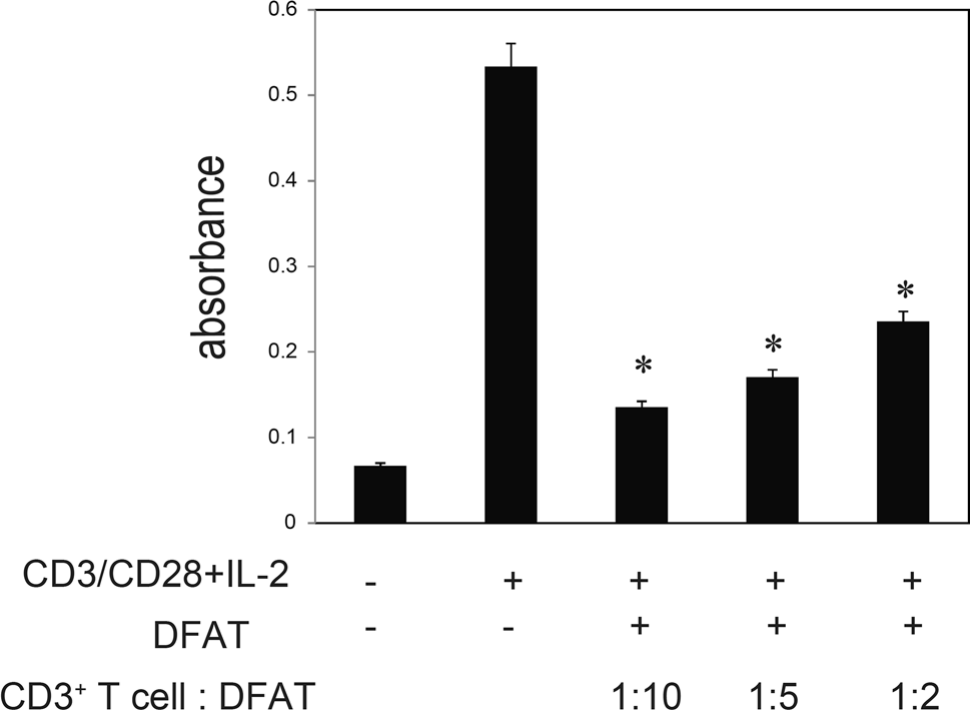
The impact of mouse DFAT cells on CD3^+^ T cell proliferation. CD3^+^ T cells isolated from mice spleens were stimulated with anti-CD3/28 antibody and IL-2 in either the presence or absence of DFAT cells. BrdU incorporation was measured using ELISA 24 h after culture. The proliferation of mouse CD3^+^ T cells was suppressed by DFAT cells in a cell density-dependent manner. Bar: mean ± SE, each sample: *n* = 3, *P < 0.05 vs CD3/CD28 + IL2 (+) DFAT (−)

We first examined whether mouse DFAT cells possess an ability to inhibit T cell proliferation. The treatment with anti-CD3/28 antibody and IL-2 significantly increased BrdU immunoreactivity in the CD3^+^ cells, which indicates T cell proliferation (Fig. 1). The stimulatory effect was significantly (*P* < 0.05) inhibited when these cells were cultured alongside DFAT cells. This relationship occurred in a DFAT cell density-dependent manner. These results indicated that DFAT cells are capable of inhibiting T cell proliferation in vitro.

### Expression of immunosuppressive genes in mouse DFAT cells

**Fig. 2:**
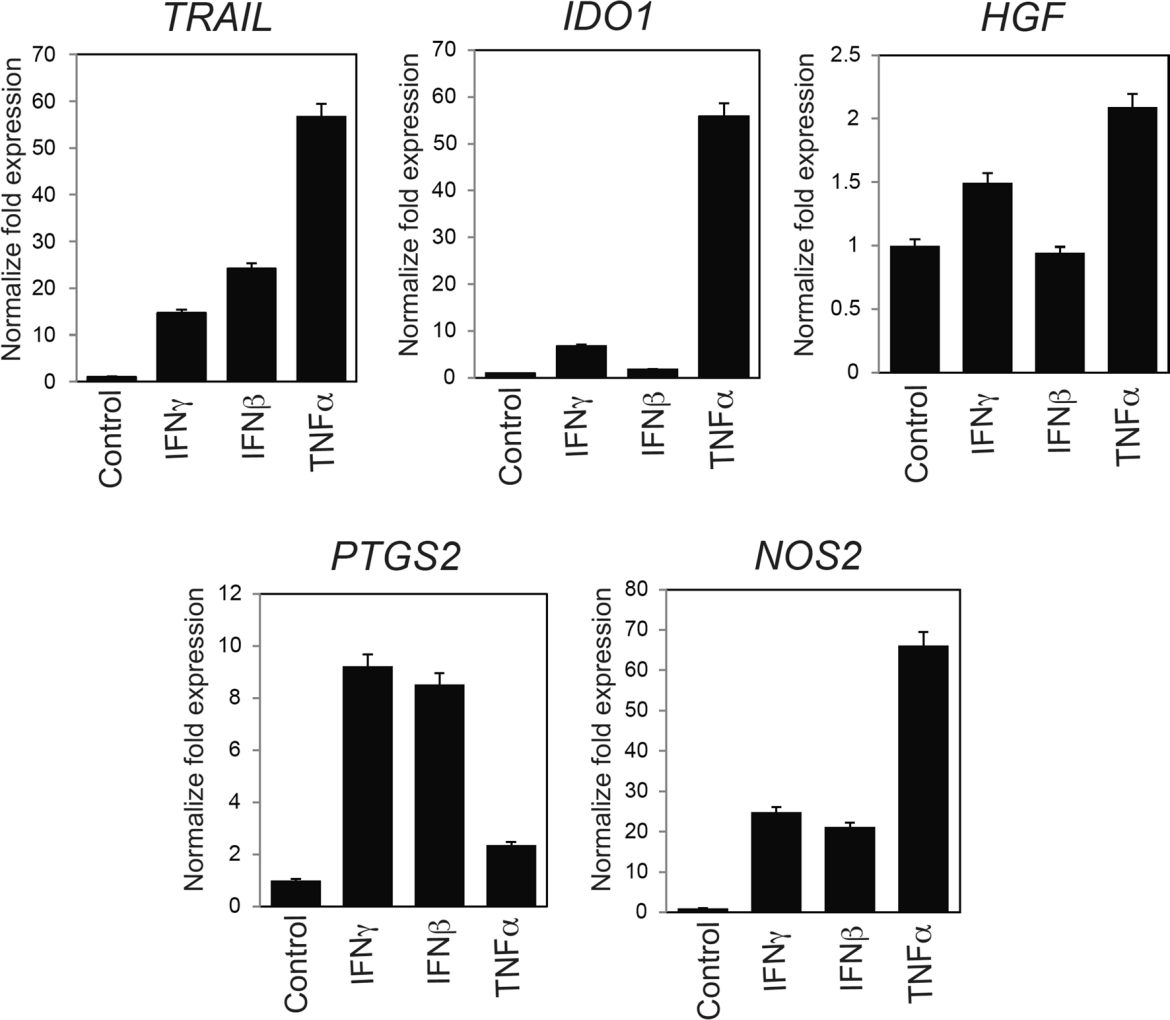
Expression analysis of immunosuppression-related genes in mouse DFAT cells. Mouse DFAT cells were stimulated with IFNγ (30 IU/ml), IFNβ (750 IU/ml), or TNFα (10 ng/ml) for 48 h. Total RNA was then extracted and subsequently *TRAIL*, *IDO1*, *HGF*, *PTGS2*, and *NOS2* mRNA were quantitated using real-time RT-PCR. Relative expression was analyzed using the comparative Ct method. *GAPDH* was used as the internal control. Expression of these genes was increased following cytokine stimulation. Bar: mean ± SD. Data shown for triplicate wells

Next, we evaluated the expression of immunosuppressive genes in response to proinflammatory cytokines in mouse DFAT cells using real-time RT-PCR. The expression of *TRAIL*, *IDO1*, *HGF*, *PTGS2*, and *NOS2* were initially ascertained in untreated DFAT cells (Fig. 2). Stimulating these cells thereafter with either IFNγ, IFNβ or TNFα increased these gene expressions in a different degree. Notably, TNFα stimulation significantly increased the expression of *TRAIL*, *IDO1*, and *NOS2* by more than 50 times as compared to the control. *PTGS2* expression was more strongly stimulated by IFNγ and IFNβ rather than TNFα. These results suggested that mouse DFAT cells possess immunosuppressive properties in response to proinflammatory conditions.

### Impact of DFAT cell transplantation on tissue damage in a mouse model of IBD

**Fig. 3:**
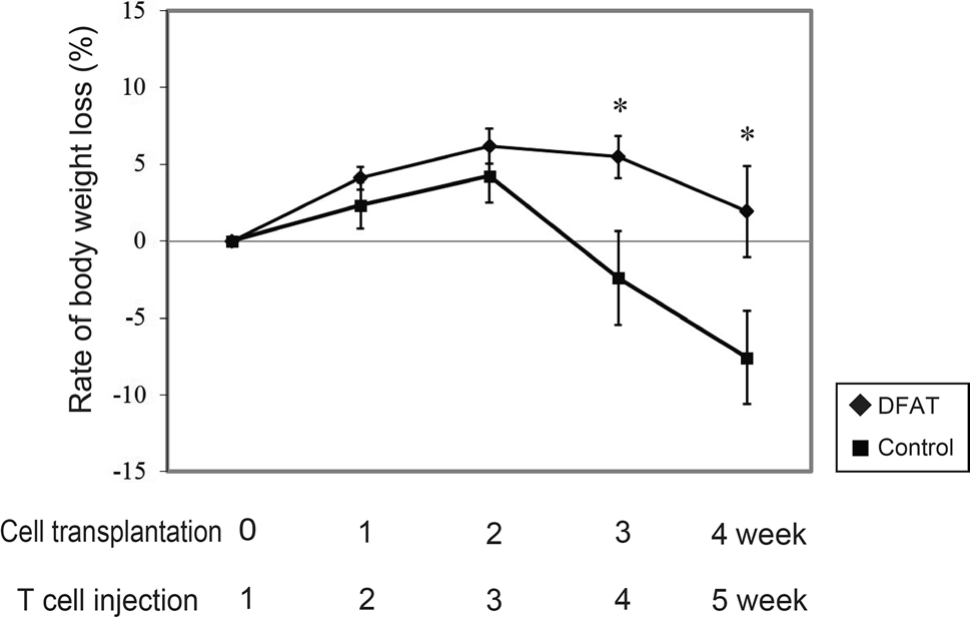
The influence of human DFAT cell transplantation on body weight in a mouse model of inflammatory bowel disease. Colitis was induced by injecting CD4^+^CD45RB^high^ T cells isolated from the spleens of BALB/c mice into the peritoneum of severe combined immunodeficiency (SCID) mice. After 1 week, either human DFAT cells (1 × 10^5^, DFAT group, *n* = 6) or saline (Control group, *n* = 6) was injected into the aforementioned mice. The body weights from each group were measured every week. The rate of weight loss in the DFAT group was significantly slower than that observed in the Control group at 3 and 4 weeks after cell transplantation. *P < 0.05 vs Control group

To further evaluate DFAT cell-based therapy in the context of IBD, we injected DFAT cells into the peritoneum of IBD mice. Our mouse model of IBD was created via adaptive transfer of CD4^+^CD45RB^high^ T cells. Weight loss was observed in the Control group at 4 and 5 weeks after T cell administration (Fig. 3). Interestingly, DFAT group significantly (*P* < 0.05) reduced the body weight loss induced by IBD when compared to the Control group at 4 and 5 weeks after T cell administration.

**Fig. 4:**
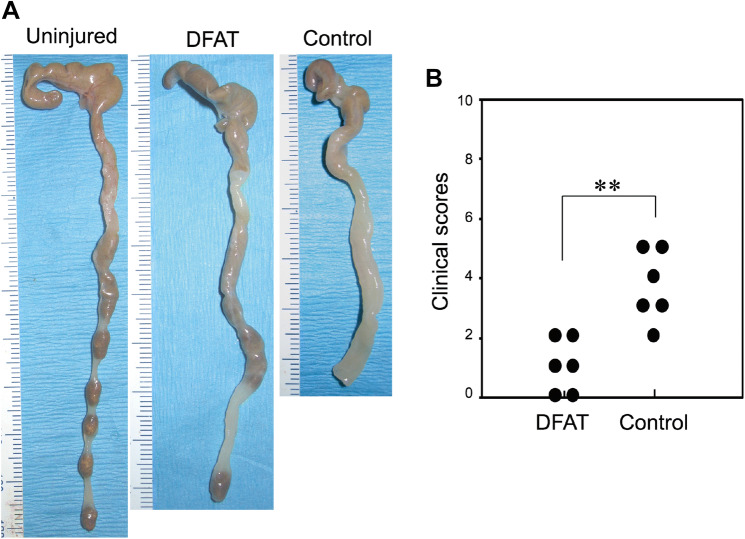
The effect of human DFAT cell transplantation on IBD clinical scores in a mouse model of IBD. The mice were sacrificed at 5 weeks after T cell administration. The colons were collected and IBD clinical scores were evaluated for each group. **a** Representative pictures of colons from each group. **b** IBD clinical scores for each group. The score for the DFAT group was significantly lower than that for the Control IBD group. **P < 0.01

**Fig. 5:**
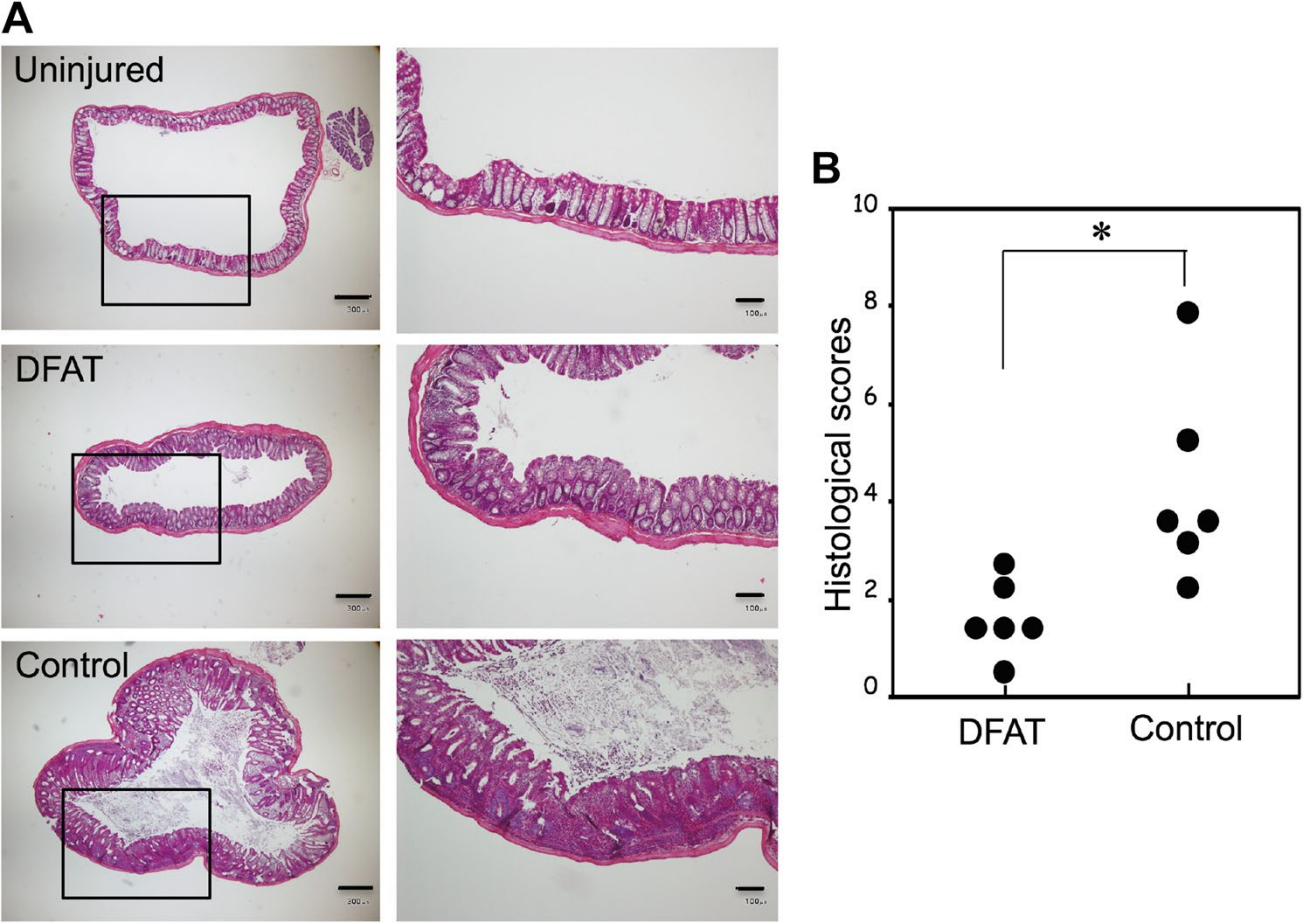
The effect of human DFAT cell transplantation on IBD histological scores in a mouse model of IBD. The mice were sacrificed at 5 weeks after T cell administration. The colons were collected and the samples were sectioned and stained with hematoxylin and eosin. **a** Representative photomicrographs of colons for each group. The right panels represent higher magnification views derived from open squares found in the left panels. Scale bar: 300 μm in the left panels and 100 μm in the right panels. **b** IBD histological scores for each group. *P < 0.05

Macroscopic analysis of the colon at 5 weeks after T cell administration revealed greater thickening of the colonic mucosa and shortening of colon length in the IBD model mice that received saline (Control group) as compared to the disease-free mice (Fig. 4a). The thickening of the colonic mucosa and shortening of colon length were both mitigated in the mice that received DFAT cell transplantation. Diarrhea and bloody stools were observed in all mice in the Control group at 5 weeks after T cell administration. However, these symptoms were rarely observed in the DFAT group. Clinical scores (hunching and wasting, colon thickening, and stool consistency) were calculated based on the macroscopic evaluation of colon injury and clinical symptoms at 5 weeks after T cell administration. Overall, the clinical score of the DFAT group was significantly (*P* < 0.01) lower than that of the Control group (Fig. 4b). Histological evaluation of injured colon 5 weeks after T cell administration demonstrated significant inflammatory cell infiltration, a reduction in goblet cells, an increase in crypt height, and destruction of crypt structure in the Control group when compared to the disease-free mice. In the DFAT group, these findings were milder than those found in the Control group (Fig. 5a). Accordingly, the histological scores (cellular infiltration, crypt elongation, and crypt abscesses) for the DFAT group were significantly (*P* < 0.05) lower than those for the Control group (Fig. 5b). As such, DFAT cell transplantation promoted recovery from tissue damage while improving clinical symptoms in the mouse model of IBD.

## Discussion

In this study, we showed that DFAT cells possessed anti-proliferative activity for T cells and expressed several immunomodulatory genes in response to inflammatory cytokines. We also found that intraperitoneally DFAT cell transplantation ameliorated colitis in the mouse T cell-transfer model of IBD. To our knowledge, this is the first study reporting the immunosuppressive ability of DFAT cells in vitro and in vivo. Our data demonstrated that coculturing CD3^+^ T cells with DFAT cells suppressed the proliferation of T cells in a cell density-dependent manner. In addition, expression of immunomodulatory genes *TRAIL*, *IDO1*, *HGF*, *PTGS2*, and *NOS2* in DFAT cells were significantly upregulated by stimulation with IFNγ, IFNβ, and TNFα. These results are similar to those from MSCs as many studies demonstrated previously [18, 25] and suggest that DFAT cells have similar immunosuppressive properties as MSCs. It was reported that TRAIL modulates T cell proliferation either indirectly by inducing immunosuppressive cells or directly by modulating T cell signaling. The latter occurs via protein tyrosine phosphorylation and nuclear translocation of the transcription factor nuclear factor-κB [26]. IDO1 converts tryptophan to the immuosuppressive metabolite kynurenine [27]. PTGS2 expression contribute to PGE_2_ production that inhibit T cell proliferation and IL-2 production [28]. Additionally, NOS2 suppresses Stat5 phosphorylation and inhibits T cell proliferation [29].

In the mouse T cell-transfer model of colitis used in the present study, the inflammation was characterized by accumulation of Th1 and T17 cells in colonic lamina propria and mesenteric lymph nodes with overexpression of INFγ and TNFα [30]. Our data showed that the DFAT cell transplantation improved weight loss, clinical scores, and histological scores in the mouse model of IBD. These findings are similar to those of previous reports indicating that MSCs suppressed intestinal inflammation in animal models of IBD [31]. Although the mechanisms how DFAT cells attenuate the experimental colitis have not been clarified, our in vitro data suggest that the transplanted DFAT cells exhibited therapeutic effect by suppressing T cell activity through an increased secretion of immunomodulatory factors such as TRAIL, IDO1, and PGE_2_ under inflammatory conditions with high concentrations of TNFα and IFNγ. The putative mechanism is supported by a previous report that intraperitoneally administration of umbilical cord MSCs improved dextran sulfate sodium (DSS)-induced experimental colitis through increased secretion of PGE_2_ [32]. In addition, it was reported that an anti-inflammatory cytokine TNFα-stimulating gene 6 (TSG-6) released from intraperitoneally injected MSCs play a role to improve IBD through polarization macrophages toward M2 phenotype [33]. Since DFAT cells have been shown to secrete TSG-6 [34], similar mechanisms of ameliorate IBD can be supposed by DFAT cell injection.

To date, clinical trials of MSCs in treatment of fistulizing and luminal IBD have been carried out in several countries. Intrafistular administration of MSCs resulted in promising beneficial effects in patients with perianal fistula associated Crohn’s disease [35]. On the other hands, therapeutic effects of MSCs on luminal IBD by systemic administration are controversial [17]. The inefficiency could be explained by findings that most of MSCs were trapped in the lungs after intravenous administration [36]. In this regard, we showed that intraperitoneally administration of DFAT cells clearly improved the effector T cell-mediated luminal IBD, suggesting intraperitoneally injection is a potent delivery method to provide the cells efficiently. To support this, previous reports demonstrated that intraperitoneal injection of MSCs showed higher cell engraftment at inflamed colon and better mucosal healing compared to intravenous injection in the DSS-colitis [37] and trinitrobenzene sulfonic acid (TNBS)-induced colitis [38]. These findings suggest that transplanted MSCs can directly interact with various immune cells in the inflamed colon. Indeed, the cell trafficking analysis revealed that intraperitoneal injected MSCs accumulated at the peritoneal lining of the inflamed colon and distributed throughout the colon wall including lamina propria and epithelial layer in the TNBS-induced colitis, and they could be detected at least 72 h after injection [38]. Similar cell mobilization and homing process might occur after intraperitoneal injection of DFAT cells.

Several characteristics of DFAT cells make them well suited for treating IBD. First, a DFAT cells can be prepared from a very small amount of fat tissue. We demonstrated that enough number of DFAT cells for transplantation was obtained within a few passages from approximately 500 mg of fat tissue [21, 39]. The large quantity of cells required for therapeutic applications is a critical hurdle impeding the clinical translation of MSCs in the treatment of immune disorders including luminal IBD. This property suggests that enough number of cells can be prepared using minimally invasive surgical or liposuction procedure. Second, DFAT cells are more homogeneous compared to MSCs [21, 39]. Bone marrow or other tissue-derived MSCs are prepared by expanding a small pool of stem cells from a population of heterogeneous cells. When culturing these cells, certain types of mature cells often contaminate the MSC populations [40, 41]. Several passages are, therefore, usually required to remove the contamination [42, 43]. Since DFAT cells are prepared from floating adipocyte fraction containing almost no other type of cells, the cells are relatively homogeneous even at relatively early passages. This property may contribute to higher safety and efficacy for future clinical applications. Our study has certain limitations. At this stage, the mechanisms how DFAT cell transplantation attenuate the colitis including tissue distribution and engraftment period has not been clarified. Also, we did not compare the therapeutic effect of DFAT cells with that of the other type of cells such as MSCs. Further studies are needed to evaluate the impact of DFAT cell transplantation on improving IBD.

## Conclusions

In summary, the current study is the first to demonstrate that DFAT cells possessed anti-proliferative potential for T cells and DFAT cell transplantation ameliorated both clinical and histopathological severity of colitis in the mouse T cell-transfer model of IBD. Because adipose tissue is recognized as easily accessible and generally abundant at any age, we propose DFAT cell administration as an attractive future therapeutic strategy for the treatment of patients with IBD.
